# Deer impact seedbanks and plant communities over 18 years of post-agricultural succession

**DOI:** 10.1371/journal.pone.0339466

**Published:** 2025-12-23

**Authors:** A. Sophie Westbrook, Aleah Butler-Jones, Scott H. Morris, Max Goldman, Anurag Agrawal, Antonio DiTommaso

**Affiliations:** 1 Department of Agronomy, Kansas State University, Manhattan, Kansas, United States of America; 2 Section of Horticulture, School of Integrative Plant Science, Cornell University, Geneva, New York, United States of America; 3 Section of Soil and Crop Sciences, School of Integrative Plant Science, Cornell University, Ithaca, New York, United States of America; 4 Department of Ecology and Evolutionary Biology, Cornell University, Ithaca, New York, United States of America; University of Udine: Universita degli Studi di Udine, ITALY

## Abstract

White-tailed deer (*Odocoileus virginianus*) are known to alter the composition of plant communities and the course of secondary succession. We present 18 years of data from a long-term deer exclosure experiment near Ithaca, NY, USA, to evaluate seedbank dynamics and the resulting aboveground community. Seedbank data were collected annually from 2005 to 2021 through a germination assay in which 280,674 emerged seedlings were identified. Aboveground data were collected from 2019 to 2022. Across all years, deer increased Shannon-Wiener diversity of seeds germinated from the soil by 8% relative to exclosure plots but did not affect abundance or species richness. Deer altered seedbank composition but not the percentage of biennial/perennial (vs. annual) or native (vs. non-native) species in the seedbank. Deer had little effect on aboveground plant communities (total cover, diversity, percentage biennial/perennial, percentage native) in 2019 or 2022. Nonetheless, deer reduced the abundance of woody plants by 50% and only the exclosure treatment had trees exceeding 1 cm diameter at breast height after 16 years. Although seedbank and aboveground plant communities changed over the study period, there was generally no interaction between the effects of exclosure treatment and year. Overall, our findings show that deer increased seedbank diversity, altered seedbank community composition, and suppressed woody plants aboveground. These changes are likely to impact the process of secondary succession. Increased seedbank diversity might increase the feasibility of passive restoration, whereas suppression of woody plants would delay forest recovery.

## Introduction

Large terrestrial herbivores represent a major influence on plant successional dynamics. By removing aboveground plant biomass with preference for certain species, they may delay, accelerate, or alter the trajectory of secondary succession [1–6]. In addition to direct effects of herbivory, large terrestrial herbivores also affect plant communities through other mechanisms including changes to nutrient cycles [7–9] and seed transport [10–13]. The effects of herbivores on plant communities depend on the environmental context, such as precipitation, temperature, nutrient availability, and disturbance regimes [14–17].

In much of the eastern United States, white-tailed deer (*Odocoileus virginianus*) are the largest herbivore and exert major influences on plant communities. Factors including land use changes, low predation risk, and reduced hunting have increased white-tailed deer populations over the last century [18–20]. Given that deer serve as keystone species in this region, changes in their abundance have far-reaching implications [21–23] for both woody and herbaceous plants [24,25]. Among woody plants, deer can reduce tree seedling density, modify the representation of different size classes, alter species composition, and increase the prevalence of introduced species [26,27]. However, effects of deer on woody plant regeneration vary in magnitude and are species- and site-specific [28,29]. Deer also modify communities of herbaceous plants in several ways [26,30]. For example, in forested ecosystems, effects of deer on herbaceous plant communities include browsing as well as indirect effects mediated by changes to the understory environment, such as increased light availability [31]. A combination of both palatable and unpalatable species may be affected [32].

In addition to modifying aboveground plant communities, deer may have long-term impacts by altering soil seedbanks. For example, seedbanks play an important role in plant recruitment, especially of early-successional species, and can serve as a buffer against plant community change [33–35]. In some cases, though not all, existing seedbanks can facilitate passive restoration after a plant community is disturbed [36,37].

Deer affect seedbanks by modifying seed rain, dispersal, and patterns of germination [17,38–41]. A survey of a suburban forest in Maryland, USA suggested that the severity of deer browsing was negatively associated with seedbank abundance and species richness [42]. A long-term experiment in West Virginia, USA found that deer presence interacted with fire and canopy gaps to support high seed bank diversity [43]. In an experiment in the southeastern New York, USA, deer did not affect the abundance, species richness, or species composition of the soil seedbank [39]. Few other studies have addressed the impacts of deer on seedbank development in the eastern United States. Given that direct effects of deer on aboveground plant communities are likely to emerge in the short-term, while effects on the seedbank may take decades (and disturbance) to be realized, an important approach is to address these effects simultaneously and to consider the timing and trajectory of impacts on both aboveground communities and seedbanks.

We began a long-term deer exclosure experiment in 2005 with pairs of exclosure and deer-browsed control plots in abandoned agricultural fields near Ithaca, NY, USA. Seedbank data were collected yearly with greenhouse germination assays and aboveground plant community data were collected in certain years. Six years of seedbank data (2005–2010), three years of aboveground plant cover data (2006–2008), and six years of woody stem counts (2005–2009, 2012) were reported previously [38]. Here we extend our previous analysis by presenting 11 additional years of data collected from the same experiment. We sought to address whether long-term trends in exclosure and control plots match the shorter-term trends reported previously. Additionally, given that several of the plots now have trees, this article includes additional information about woody species in the seedbank and the number, species composition, and size of trees growing in the plots.

By extending our long-term dataset from eight to 18 years, we were able to test hypotheses about the persistence of deer effects and the temporal dynamics of change during secondary succession. We tested three main hypotheses, as follows.

### Hypothesis 1

Effects of deer on the seedbank previously reported by DiTommaso et al. [38] continue a decade later, but the magnitude of these effects will decrease over time. In the first few years of this experiment, deer reduced abundance and species richness, increased Shannon-Wiener diversity, increased the percentage of annuals, and reduced the percentage of native species in the seedbank. We expected the magnitude of these effects to decrease over time because the plots have by now been dominated by perennials for well over a decade and the percentage of native species is increasing. According to our hypothesis, initial dynamics dominated by colonizing species may persist only early in the successional process and as long as those colonizing species’ seeds persist in the soil.

### Hypothesis 2

For the aboveground community, the primary effect of deer will be suppression of woody species. We previously observed a suppressive effect of deer on establishment of woody species after eight years [38]. The progress of secondary succession has substantially advanced after 18 years without a major disturbance, providing the opportunity to assess the effects of deer on the next stage of succession. Belowground, we hypothesized that the seedbank of woody species would not be affected by deer browsing because these seeds are not generally persistent in the soil but are rather dispersed by wind or animals.

### Hypothesis 3

Each of the evaluated parameters may reach a plateau by the end of our 18-year data set. This temporal trend was expected to occur in both treatments (exclosure and control) and be driven by the plots entering the next successional stage. This hypothesis does not address expected differences between the exclosure and control treatments (Hypotheses 1–2 above) but instead predicts that the rate of change over time will be slowing for all parameters and treatments.

## Materials and methods

Characteristics of the study site, experimental design, seedbank data collection, and aboveground vegetation survey were previously reported by DiTommaso et al. [38]. A summary of this information is provided below. The current article also reports on a new woody plant survey. No permits were required to carry out this research, which involved no direct handling of deer.

### Study site and experimental design

In 2005, six blocks were established within abandoned agricultural fields near Ithaca, NY, USA (42°27’, 76°26’W). Soils are poorly drained and mostly silt loams, including Erie Channery silt loam and Erie-Ellery Channery silt loam [44]. The total precipitation normal for this area (1991–2020) is 97 cm and the mean average temperature normal is 8 C [“Ithaca Cornell University” station, 45].

While all sites were historically used for agricultural production, their recent uses varied [38]. Blocks 1 and 2 had been fallow without tillage for at least 23 years before the experiment. Block 3 was a hay field that had been fallow and occasionally mowed for five to six years. Blocks 4, 5, and 6 were primarily hay fields that had also been used for corn production. All blocks, except for Block 5, are within 20 m of a forest’s edge. Block 5, however, is located approximately 100 m from forest. To reduce the impact of variation in land use history across blocks, each block was mowed, treated with label-recommended rates of glyphosate, and tilled using a disk harrow prior to starting the experiment.

Two 15 by 15-m plots were established in each of the six blocks. The two plots in each block were randomly assigned to two treatments: a deer-browsed control treatment in which one plot is left accessible to deer, and an exclosure treatment in which deer are not able to access the plot. The exclosure plot within each block is surrounded by 2.5 m tall, woven wire fences. This fencing prevents deer from entering the plot while allowing smaller animals and birds to freely enter and exit the plot.

### Seedbank data collection

From 2005 to 2021, soil samples were collected in late October each year. For this sampling, each plot was divided into four equally sized subplots. Soil samples were taken using an 8 cm-diameter soil corer from the top 15 cm of the soil profile. Twelve soil samples were collected from each subplot, homogenized to a final volume of 3.5 L, bagged, and stored at 4 C for at least six weeks (sometimes longer). It is likely that this period of cold stratification reduced physiological dormancy in most species, but we acknowledge that a longer period of stratification might have further reduced dormancy. After this initial cold stratification period, each 3.5 L soil sample was mixed in a 2:1 ratio with a 0.2 m^3^ peat, 0.2 m^3^ vermiculite, and 0.1 m^3^ perlite plus 1.8 kg CaSO_4_, 1.8 kg 10–5–10 N-P-K, and 2.3 kg lime. Any sizeable roots, rhizome fragments, or rocks were removed, then the mixture was spread in plastic flats to a depth of 2.5 cm. In total, 48 flats were prepared each year: 24 flats from exclosure plots and 24 flats from deer-accessible plots.

Flats were placed in a greenhouse set to a 14-h day length and 24/21 C day/night temperature regime. Flats were watered when necessary and fertilized weekly with 200 ml of a 21-5-20 N-P-K solution. Over seven weeks, the flats were examined on a regular basis for the presence of newly emerged seedlings. Seedlings were identified to the species once an accurate identification was possible, counted, and removed from the flat. After this seven-week period, flats were placed back into a cooler and stored at 4 C for an additional three weeks of cold stratification. After three weeks, the soil in each flat was disturbed to redistribute seeds within the soil profile and flats were returned to the greenhouse for a second seven-week period. The greenhouse conditions and counting procedure were the same as in the first germination cycle.

Emerged seedling counts were summed across the four flats (representing four subplots) for each plot to create a single total of emerged individuals per species per plot. This total was used for analysis (i.e., the experimental unit is the plot rather than the subplot).

*Oxalis stricta* L. (yellow woodsorrel) and *Oxalis corniculata* L. (creeping woodsorrel) were not always reliably distinguished in the seedbank emergence assay, so were treated as a single species for most analyses and excluded from the life cycle analysis. Both *Oxalis* spp. and *Clinopodium vulgare* L. (wild basil; unknown native range) were excluded from the analysis of native versus introduced seedbank species.

### Aboveground data collection

In August–September 2019 and August 2022, we collected data on aboveground plant cover. Data were collected in the four quadrants (northeast, northwest, southeast, and southwest) of each plot. In each quadrant, we placed a 1 by 1-m quadrat and visually estimated the ground cover of each species that was present.

In addition to the whole-community measurements, we took additional measurements on *Solidago* spp. because they dominated aboveground plant communities at this successional stage. During our survey in 2019, we measured the biomass of *Solidago* spp. (goldenrods) within each quadrat. *Solidago* spp. biomass was clipped at ground level, dried at 60 C to a constant weight, and weighed. In 2021, *Solidago* spp. plant height was measured within the interior 100 m^2^ of the plot. The dimensions of the 100 m^2^ sampling area were 10 by 10 m, leaving a 2.5 m buffer at the edges of the 15 by 15 m plot. One *Solidago* spp. height measurement was collected within each quadrant of this area, for a total of four *Solidago* spp. height measurements per plot.

For aboveground plant cover of each species, *Solidago* spp. biomass, and *Solidago* spp. height, we collected four measurements per plot (one per quadrant). In each case, we calculated the mean of the four measurements to produce a single average value per species per plot. This average value was used for analysis (i.e., the experimental unit is the plot rather than the subplot).

In the aboveground whole-community set, *Carex* spp. (unknown sedge) was excluded from the diversity analysis because this unknown sedge may have been the same as sedges that were identified to species. In 2019, *Clinopodium vulgare* was again excluded from the native/introduced analysis due to uncertainty about its origin. In 2022, *Carex* spp. was excluded from the native/introduced analysis.

### Woody plant survey

For the woody plant survey performed in 2021, six 10-m transects were defined within the interior 100 m^2^ sampling area of each plot. There were 2 m between transects and a 2.5 m buffer between transect ends and plot edges. The abundance of woody plants was assessed by counting all woody plants along the transect line ±15 cm to either side of the tape (10 m transect length by 0.3 m width by six transects = 18 m^2^). We also counted and identified the number of woody plants reaching above the herbaceous plant (usually *Solidago* spp.) canopy within the entire 100 m^2^ sampling area. Diameter at breast height (DBH) was measured for plants within the sampling area that exceeded 1 cm DBH.

### Statistical analysis

Data analyses were performed in R version 4.3.1 [46]. Univariate analyses for the seedbank data were performed using piecewise linear mixed models (package *lme4*). Piecewise mixed models were used instead of generalized linear (e.g., logistic) mixed models because they enabled us to distinguish initial trends in the first few years following field abandonment from long-term trends. Piecewise models were fitted for the following seedbank response variables: abundance of the whole community, abundance of three individual taxa [*Rumex crispus* L. (curly dock), *Oxalis* spp. (woodsorrel), and *Panicum capillare* L. (witchgrass)], species richness, Shannon-Wiener diversity (not exponentiated), biennial and perennial species as a percentage of abundance or richness, and native species as a percentage of abundance or richness. This modeling approach was appropriate for the percentage data because our percentage variables never approached the boundaries of 0% or 100%. Instead, the percentage data were distributed in the middle of the range in a roughly normal fashion so that all model diagnostics were met without requiring transformation.

For each model, fixed effects were (1) time prior to a breakpoint (that is, a certain year within the study period), (2) time following the breakpoint, (3) deer exclosure treatment, (4) interaction between deer exclosure treatment and time prior to the breakpoint, and (5) interaction between deer exclosure treatment and time following the breakpoint. Random effects were block and unique plot (combination of block and deer exclosure treatment). The breakpoint was selected by fitting the model for each possible breakpoint (i.e., year within the study range) and choosing the value that resulted in the lowest model AIC.

Response variables were sometimes transformed to meet model assumptions (normality and equal variance of residuals). In the seedbank data set, we transformed total abundance (natural logarithm), *R. crispus* abundance (1/4 power), *Oxalis* spp. abundance (1/4 power), and *P. capillare* abundance (1/3 power). These transformations reflect the structure of plant abundance data, which tend to be distributed with a long right tail and variance increasing with the mean. After transformation, all model assumptions were met. Significance was assessed with Type III ANOVA.

To evaluate seedbank community composition, we performed a PERMANOVA evaluating the impacts of deer exclosure treatment and year (package *vegan*). Permutations were carried out randomly within combinations of block and treatment. The number of permutations was 999 and the Bray-Curtis method was used to calculate pairwise differences. The Bray-Curtis method was also used to calculate pairwise differences for the nonmetric multidimensional scaling (NMDS; function *metaMDS* calling *monoMDS* within package *vegan*). For the NMDS, the data transformation was a Wisconsin double standardization on the square root of abundance data. Indicator species analyses were performed across all years and individually for each year (package *indicspecies*). Indicator values were calculated using the IndVal.g method considering both the frequency and the abundance of species within each group.

The aboveground vegetation survey data from 2019 and 2022 were analyzed separately. We used linear mixed models with deer exclosure treatment as a fixed effect and block as a random effect. Response variables were total plant cover, species richness, Shannon-Wiener diversity, percentage of biennial/perennial species, percentage of native species, and goldenrod biomass (2019 only). The same model structure was used to evaluate woody plant abundance, woody species richness, *Solidago* spp. height, and the number of woody plants above the *Solidago* spp. layer in 2021. We also created linear mixed models testing the effects of the 2018 seedbank (total cover, species richness, or Shannon-Wiener diversity), deer exclosure treatment, and the interaction, with block as a random effect, on the 2019 aboveground vegetation (total cover, species richness, or Shannon-Wiener diversity). The same structure was used to test effects of the 2021 seedbank, deer exclosure treatment, and the interaction on the 2022 aboveground vegetation.

We applied square root transformations to most of the aboveground response variables. The exceptions were species richness and Shannon-Wiener diversity in 2019 and 2022 (no transformation needed) and *Solidago* spp. height in 2021 (natural logarithm). After transformation, all model assumptions were met. Significance was assessed with Type III ANOVA.

## Results

### Seedbank abundance and diversity

For all piecewise linear mixed models (univariate seedbank analyses), we report on five fixed effects: (1) time prior to a breakpoint (that is, a certain year within the study period), (2) time following the breakpoint, (3) deer exclosure treatment, (4) interaction between deer exclosure treatment and time prior to the breakpoint, and (5) interaction between deer exclosure treatment and time following the breakpoint.

**Fig 1:**
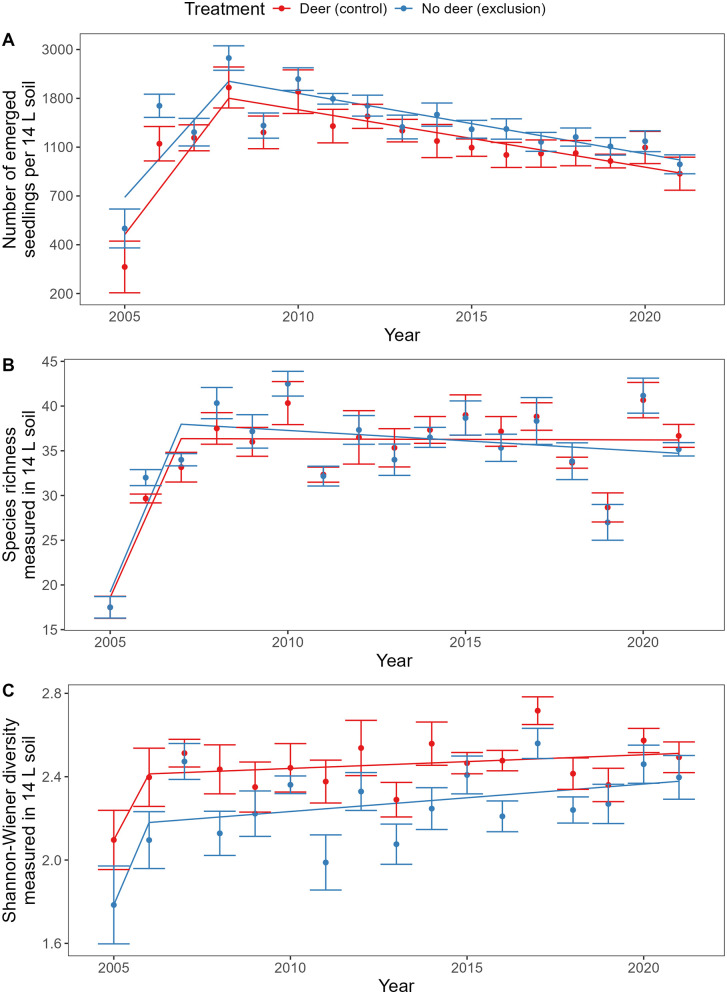
Seedbank (A) abundance, (B) species richness, and (C) Shannon-Wiener diversity based on greenhouse germination assay. Mean ± 1 standard error across six blocks, alongside fitted model described in the text. A total soil volume of 14 L was collected from each plot and all data are presented on this scale. For abundance, data were modeled on a logarithmic scale but the y axis is relabeled to show abundance on the original scale.

Seedbank abundance, evaluated as the number of seedlings emerged in greenhouse assays, increased from 2005 to 2008 (F_1,188_ = 210, P < 0.001), then decreased by 64% over the following 13 years (F_1, 188 _= 120, P < 0.001; Fig 1A). Across the entire study period, deer reduced seedbank abundance by 14%, relative to the exclosure plots, but this effect was marginally significant (F_1, 29 _= 3.4, P = 0.075). There were no interactions between deer exclosure treatment and year (prior to 2008, F_1,188_ = 1.4, P = 0.24; after 2008, F_1,188_ = 0.076, P = 0.78).

**Fig 2:**
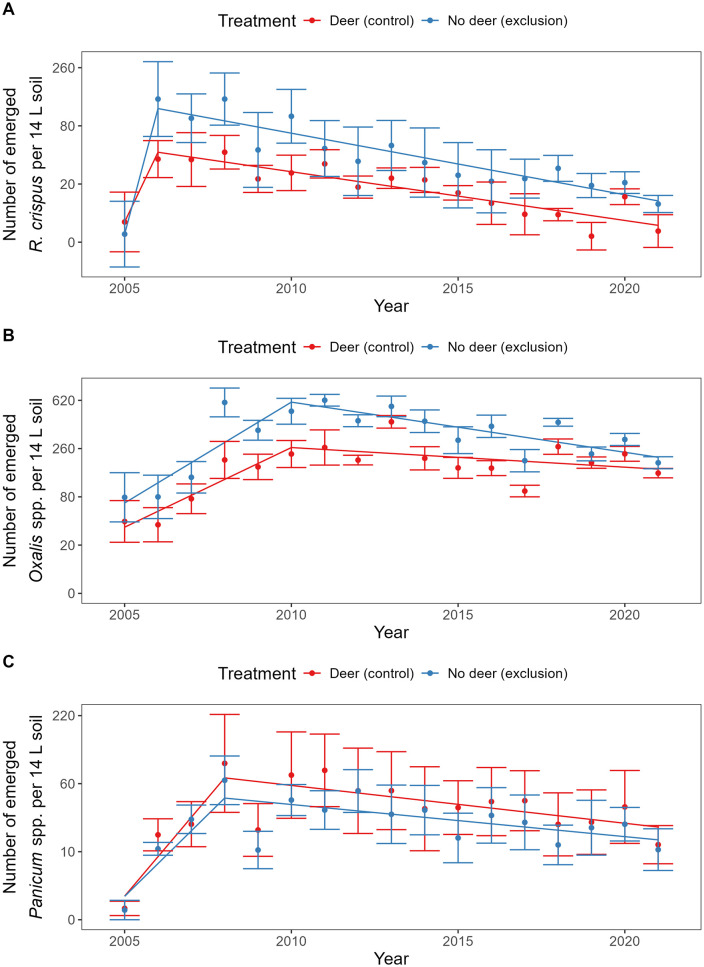
Effects of treatment and year on seedbank abundance of (A) *Rumex crispus*, (B) *Oxalis* spp., and (C) *Panicum capillare.* Mean ± 1 standard error across six blocks, alongside fitted model described in the text. A total soil volume of 14 L was collected from each plot and all data are presented on this scale. Data were modeled on transformed scales (*R. crispus* and *Oxalis* spp.: ¼ power; *P. capillare*: 1/3 power) but the y axes are relabeled to show abundances on the original scale.

There were 144 species identified in the seedbank analysis. Most were relatively rare: 75 species had fewer than 100 emerged seedlings in the greenhouse assay and 103 species had fewer than 500 emerged seedlings. However, seven species had more than 10,000 emerged seedlings (Table 1). Of these seven species, *R. crispus* was strongly underrepresented in browsed control plots, with only 22% of emerged seedlings of this species occurring in control plots (Fig 2A). By contrast, control plots accounted for 46% of emerged seedlings across all species. *Rumex crispus* seedlings were significantly more abundant in the exclosure plots (F_1, 9 _= 5.4, P = 0.046) and showed a greater increase between 2005 and 2006 (F_1, 188 _= 5.0, P = 0.027). *Oxalis* spp. (predominantly *O. stricta*) were also underrepresented in control plots (35% of the total for this species; Fig 2B). These seedlings were significantly more abundant in the exclosure plots (F_1, 15 _= 22, P < 0.001) but abundance decreased more rapidly in exclosure vs. control plots after 2010 (F_1, 188 _= 7.0, P = 0.0088). On the contrary, *P. capillare* was numerically overrepresented in control plots (69% of the total for this species; Fig 2C). This difference between control and exclosure was not significant (F_1, 7 _= 0.72, P = 0.42). For each of these three species, abundance initially increased (positive effect of year prior to breakpoint in the piecewise model, P < 0.05) and subsequently decreased (negative effect of year after the breakpoint, P < 0.05). The breakpoints occurred in 2006 for *R. crispus*, 2010 for *Oxalis* spp., and 2008 for *P. capillare* (Fig 2).

**Table 1:** Most abundant species observed in seedbank germination assay between 2005 and 2021. Approximately 2,856 L of soil were germinated over the duration of this experiment.

Common name	Scientific name	Native^1^	Abundance in browsed control treatment	Abundance in exclosure treatment	Percentage in control
Woodsorrel	*Oxalis* spp.	N	20938	38629	35
Yellow rocket	*Barbarea vulgaris* W.T. Aiton	I	19659	16695	54
Tall goldenrod	*Solidago altissima* L.	N	11278	12822	47
White campion	*Silene alba* (Mill.) Krause	I	7367	10564	41
Path rush	*Juncus tenuis* Willd.	N	8040	8219	49
Witchgrass	*Panicum capillare* L.	N	9806	4324	69
Curly dock	*Rumex crispus* L.	I	2757	9858	22
All 144 species			130124	150550	46

^1^Native (N) or introduced (I) to New York, USA.

Seedbank species richness increased from 2005 to 2007 (F_1, 188 _= 160, P < 0.001) but was unaffected by year after 2007, exclosure treatment, or interactions between year and exclosure treatment (Fig 1B). In contrast, Shannon-Wiener diversity increased rapidly from 2005 to 2006 (F_1, 188 _= 22, P < 0.001), then more slowly from 2006 to 2021 (F_1, 188 _= 7.1, P = 0.0083; Fig 1C). Deer increased overall Shannon-Wiener diversity by 8% relative to exclosure plots (F_1, 24_ = 9.5, P = 0.0052).

### Seedbank community composition

Overall, woody species accounted for only 47 of the 280,674 seedlings germinated in the seedbank assay. These 47 seedlings belonged to six species: four trees, a shrub, and a woody vine (S1 Appendix). There was no apparent difference in the abundance of woody species between the browsed control and the exclosure treatment (S1 Appendix).

**Fig 3:**
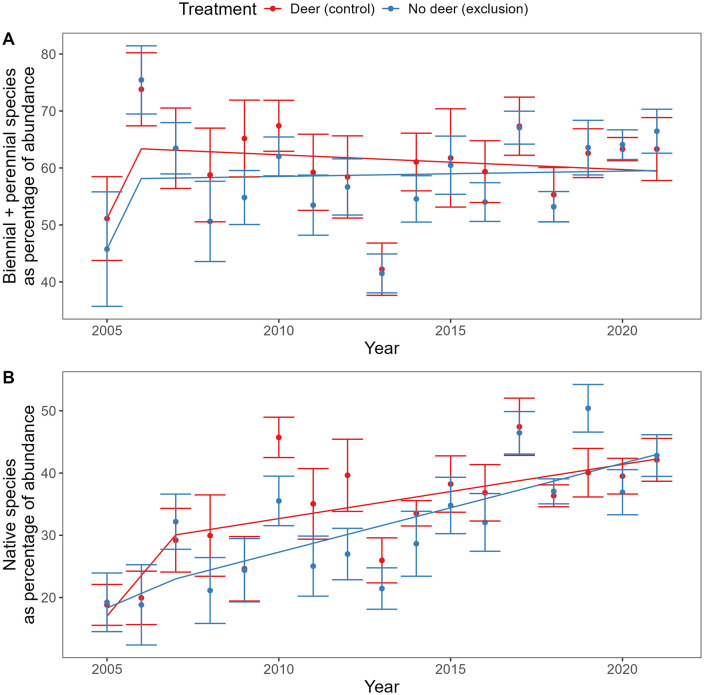
Percentage of total seeds that were (A) biennial or perennial and (B) native in a greenhouse germination assay. Mean ± 1 standard error across six blocks, alongside fitted model described in the text.

The prevalence of biennial or perennial seeds, as a percentage of seedbank abundance, initially increased from 2005 to 2006 (F_1, 188 _= 11, P = 0.0011). Otherwise, effects of year and deer exclosure treatment were not significant (Fig 3A). The prevalence of native seeds, as a percentage of seedbank abundance, increased from 2005 to 2007 (F_1, 188 _= 8.7, P = 0.0036) and from 2007 to 2021 (F_1, 188 _= 48, P < 0.001; Fig 3B). Early in the study, there were non-significant trends towards greater relative abundance of biennial/perennial and native seeds in the control treatment browsed by deer, but these trends later disappeared (Fig 3).

The prevalence of biennial and perennial species, as a percentage of seedbank species richness, increased with year (F_1, 190 _= 84, P < 0.001) but was not significantly affected by deer exclosure treatment (S2 Appendix). There was a non-significant positive effect of deer on the relative richness of biennial and perennial species. The prevalence of native species, as a percentage of seedbank species richness, increased rapidly from 2005 to 2008 (F_1, 188 _= 110, P < 0.001) and more slowly from 2008 to 2021 (F_1, 188 _= 8.0, P = 0.0051; S2 Appendix). Deer did not impact the percentage of native species (F_1, 40 _= 0.83, P = 0.37).

**Fig 4:**
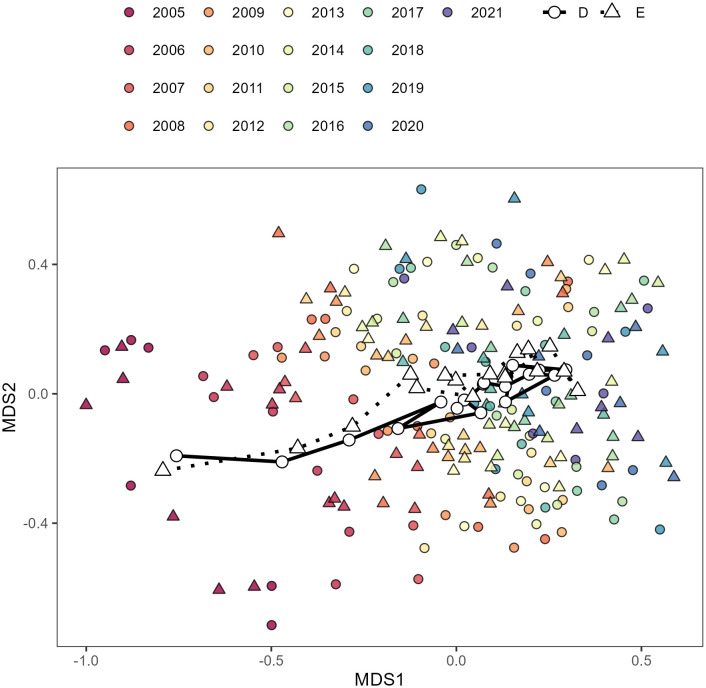
Non-metric multidimensional scaling (Bray–Curtis) based on seedbank abundance. There are 204 points shown (two treatments by six blocks by 17 years). Point shape represents treatment (circle for “D”, deer-browsed control, and triangle for “E”, exclosure) and point color represents year (beginning with 2005 in red). The open shapes represent the centroids for each combination of treatment and year. The centroids for all 17 years within each treatment are connected by paths (solid for the deer-browsed control and dotted for the exclosure treatment).

PERMANOVA revealed strong effects of deer exclosure treatment and year on seedbank composition (F_17 _= 4.2, P = 0.001). PERMANOVA was also significant when exclosure treatment (F_1_ = 6.8, P = 0.034) and year (F_16_ = 3.8, P = 0.001) were tested separately. The NMDS showed separation between the first few years of the study and later years in both treatments (Fig 4). The indicator species analysis including all years identified 36 of 144 species that were associated with either control or exclosure treatments (S3 Appendix). Of these 36 species, 14 species, including eight biennial/perennial species and four native species, were associated with the control treatment. The remaining 22 species, including 12 biennial/perennial species and 10 native species, were associated with the exclosure treatment. Despite these general trends, none of these associations were significant (P > 0.05). Only *Dactylis glomerata* L. (orchardgrass), associated with the control treatment, was close to significance (P = 0.06).

When indicator species analyses were performed separately by year, the number of identified species increased over time from 4 species in 2005 to 38 species in 2021. This finding suggests increasing divergence between the deer exclosure treatments, as also suggested by the highly significant PERMANOVA. However, associations between specific species and treatments were generally not significant, except that *Cerastium fontanum* Baumg. (mouse-ear chickweed) was significantly associated with the presence of deer in 2009. The lack of significance for individual species suggests that treatment effects were spread across many species rather than being driven by major impacts of deer on a few dominant indicator species.

### Aboveground plant community

We did not observe any effect of deer exclosure treatment on aboveground plant cover, species richness, or Shannon-Wiener diversity in either 2019 or 2022 (S4 Appendix). Deer exclosure treatment did not affect the percentage of species that were biennial/perennial or native in either year (S4 Appendix). Neither deer exclosure treatment nor 2018 seedbank characteristics (abundance, richness, or Shannon-Wiener diversity) affected the corresponding characteristics of the 2019 aboveground plant community. Similarly, neither deer exclosure treatment nor 2021 seedbank characteristics significantly affected the 2022 aboveground plant community. The 2019 *Solidago* spp. biomass and 2021 *Solidago* spp. height were not impacted by deer exclosure treatment (S4 Appendix).

**Fig 5:**
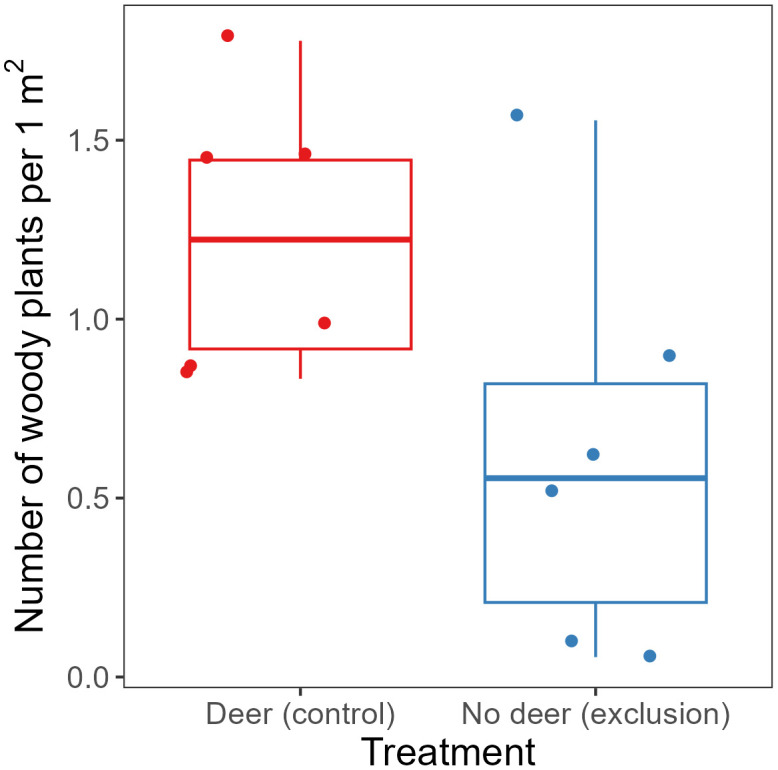
Number of woody plants counted along transects in 2021 survey. Each box represents the interquartile range (IQR) for each treatment while the whiskers indicate the range of the data within 1.5 times the IQR. Each point represents an exclosure (no deer) or deer-accessible control plot within a block. For each plot, data were collected in an 18 m^2^ area and have been rescaled to plants per 1 m^2^ by dividing by 18.

Deer reduced the abundance of woody plants by 50% (mean ± SE, 22 ± 3 woody plants per 18 m^2^ in the exclosure treatment and 11 ± 4 woody plants per 18 m^2^ in the browsed control treatment; F_1, 10_ = 5.3, P = 0.044; Fig 5). Woody species richness, assessed within the same 18 m^2^ sampling area, averaged 5.0 ± 0.6 in the exclosure treatment and 3.8 ± 1.2 in the browsed control treatment (F_1, 5_ = 1.4, P = 0.29). The number of trees overtopping the herbaceous plant canopy averaged 3.8 ± 2.0 trees per 100 m^2^ in the exclosure treatment and 1.2 ± 0.8 trees per 100 m^2^ in the browsed control treatment (F_1, 5_ = 2.3, P = 0.19). In the exclosure treatment, trees overtopping the herbaceous plant canopy included *Populus deltoides* W.Bartram ex Marshall (8, common cottonwood), *Rhamnus* spp. (4, buckthorn), *Cornus* spp. (3, dogwood), *Lonicera* spp. (3, honeysuckle), *Viburnum dentatum* L. (2, arrowwood viburnum), *Rosa multiflora* Thunb. (2, multiflora rose), and *Robinia pseudoacacia* L. (1, black locust). In the browsed control treatment, trees overtopping the herbaceous plant canopy included *R. multiflora* (3), *Platanus occidentalis* L. (3, American sycamore), and *Carya* spp. (1, hickory).

Diameter at breast height (DBH) data were only collected from trees in the exclosure treatment because no tree in the browsed control was large enough to take this measurement. In the exclosure treatment, we identified seven *Populus deltoides* trees of sufficient size, averaging 14.7 ± 2.6 cm DBH and also identified one *R. pseudoacacia* tree with 23.0 cm DBH.

## Discussion

This 18-year study demonstrated old-field succession in the presence and absence of white-tailed deer, which are the primary large herbivores in the region. Resident plant communities reflecting moderate levels of past disturbance experienced one intense disturbance at plot establishment in 2005 and were not subsequently disturbed during the study. These changes in disturbance level likely accounted for the initial rapid increase in seedbank abundance. A peak followed by a decline in seedbank abundance is a typical consequence of field abandonment [47]. This decline in seedbank abundance is consistent with the process of succession because later-successional species are less likely to form persistent seedbanks [33,35]. Also consistent with succession, we observed a steady increase in the prevalence of biennial/perennial species, relative to annual species, as a percentage of seedbank species richness across the entire study period.

Deer weakly reduced seedbank abundance. Although statistically non-significant, this trend would agree with our previous research [38]. Our finding could indicate that deer browsing did reduce seed production by palatable species, but a combination of continued seed production by other species, seed dispersal into the area, and seed persistence in the seedbank prevented a more dramatic decline in seedbank abundance. Similar to our results, Beauchamp et al. [42] reported that increased deer browsing pressure was associated with reduced seedbank abundance in a suburban secondary forest. They suggested that deer either reduced inputs to the seedbank or preferred areas dominated by later-successional species that did not form large seedbanks. In contrast, Levine et al. [39] did not find any significant association between deer browsing and seedbank abundance in hunted or non-hunted forest habitats in New York, USA. Seedbank abundance was numerically higher in a deer exclosure treatment, relative to an unfenced control, at one of the two sites [39].

The species richness of the seedbank increased for the first few years of the experiment before reaching a plateau. The initial increase was likely associated with the increase in seedbank abundance over the same period, as the absence of disturbance would enable many resident and colonizing species to produce seeds. The plateau may have occurred once most species present in the area and capable of contributing to the seedbank had done so. No effect of deer on species richness was observed. This finding likely reflects the fact that species contributing to early-successional seedbanks typically have long-lived seeds [48–50]. For species whose seeds do not accumulate or persist in the soil, seed dispersal may play an important role in maintaining their presence at a site [33,48]. In the context of seed dispersal, it is important to note that our plots were relatively small (15 by 15 m). Therefore, wind dispersal likely deposited seeds from surrounding areas throughout all plots, ensuring some representation of various species that were growing nearby. This wind dispersal is the likely source of the few woody seedlings, including tree seedlings, observed in the seedbank germination assay. Tree seeds probably dispersed into the experimental plots from neighboring forest areas. In addition, it is possible that even preferred species were occasionally missed by deer during browsing, resulting in occasional additions to the seedbank.

Deer significantly increased the seedbank Shannon-Wiener diversity index compared with the exclosure treatment [see also 38]. As seedbank species richness did not change, this trend reflected increased evenness in the browsed treatment. Deer would be likely to promote evenness if they showed a preference for dominant species, thereby reducing the dominance of these species. Consistent with this expectation, we found that deer tended to reduce the dominance of *Oxalis* spp. and *R. crispus*, which were among the most abundant taxa observed in the seedbank survey. In contrast, deer promoted abundance of *P. capillare* (Fig 2). Overall, monocot species may be less vulnerable to browsing by white-tailed deer [51].

Some previous studies have revealed negative or neutral effects of deer browsing on plant diversity in fields or forests, with negative effects becoming more likely at high browsing pressure [21,22,52–55]. In forests, tree species diversity is especially likely to be reduced by deer [56,57]. However, effects of deer on plant diversity depend on plant community composition and browsing pressure. If dominant, large, or fast-growing species are palatable, low to moderate levels of deer browsing may instead increase evenness and diversity by enabling other species to access more resources [21,58–60]. Our findings are consistent with this latter scenario, which would make sense given that palatable species accounted for a substantial portion of plant cover in the exclosure treatment. Relatively few native species were associated with the browsed control treatment, according to the indicator species analysis across all years. These data may suggest that native species were more palatable to deer [61].

In addition to browsing, another explanation for impacts of deer on diversity and functional composition could involve interspecific differences in the extent of seed transport by deer. White-tailed deer are known to be important agents of seed transport in eastern North America. For example, a study on endozoochory in New York, USA found that more than 70 plant species germinated from white-tailed deer feces [40, see also 10,62]. White-tailed deer are also known to disperse seeds through epizoochory [63]. Our study design did not enable us to distinguish between the impacts of deer browsing and seed dispersal by deer.

The aboveground vegetation survey did not reveal major impacts of deer on the herbaceous plant community. This finding may reflect a combination of methodological and ecological factors. Methodologically, we collected fewer data points on the aboveground plant community, compared with the soil seedbank. Ecologically, the soil seedbank serves as a reservoir for plant diversity and buffer against change, especially in early-successional ecosystems [33–35,64]. It is plausible that the relative stability of the soil seedbank revealed trends that were masked by greater variability in the aboveground plant community.

The aboveground woody plant survey revealed that excluding deer increased the number of woody plants and allowed woody plants to grow above the herbaceous plant canopy. Conversely, deer largely suppressed woody plants. This finding is consistent with previous research demonstrating that browsing by deer can reduce or even eliminate tree growth [6,24,25,27,30,56,65]. In the browsed treatment, only *R. multiflora* and *P. occidentalis*, plus one *Carya* spp., extended above the herbaceous plant canopy. *Rosa multiflora* is an invasive shrub and although it can be utilized by deer for food and cover [66], browsing by deer is not sufficient to control this species. Prescribed browsing by goats has been evaluated as a potentially more effective strategy than browsing by deer [67,68]. *Platanus occidentalis* can also be consumed by deer [69,70] and deer can reduce seed rain by this species [71]. However, deer browsing of *P. occidentalis* has been reported to be lighter than browsing of other tree species [72]. Overall, it appears that the effect of deer on the woody plant community was due to direct browsing on newly dispersed and germinated seeds, as there was little evidence of woody species in the seedbank.

Previous research has suggested that large terrestrial herbivores can delay, accelerate, or change the course of succession [1–5]. Our data set provides some evidence for multiple possibilities. For example, the increase in seedbank Shannon-Wiener diversity in the browsed control might be characterized as an acceleration. Changes to species composition revealed by the PERMANOVA could be consistent with an altered course of succession. In the aboveground data set, suppression of woody plants constitutes a delay of the successional process.

Our research also provides insight into questions about the role of seedbanks in ecological restoration [36,37,41,73]. Passive restoration strategies, in which ecosystems are allowed to recover from disturbance with minimal human interference, often rely on recruitment from existing seedbanks rather than reseeding. The feasibility of this approach has been debated. Especially in old fields (abandoned cropland), seedbanks are frequently dominated by early-successional species, although more desirable later-successional species may also be represented [33,47,74]. Our study is consistent with this pattern. The most abundant species in the seedbank were early-successional. However, the seedbank included a diverse community of herbaceous species, especially in the deer treatment. The effect of deer shows that herbivores modify (in this case, potentially enhance) the restoration potential of seedbanks. As expected, woody species were barely represented in the seedbank, so it is unlikely that the seedbank would contribute much to shrub and tree recruitment.

## Conclusions

In summary, our 18-year study provided insight into belowground and aboveground effects of field abandonment and white-tailed deer in the northeastern United States. Following field abandonment, the old field seedbank grew for a few years and subsequently shrank. Seedbank diversity grew for a few years before leveling off. Changes in seedbank community composition continued throughout the entire study period. Deer increased seedbank diversity and altered seedbank community composition. This effect suggests a long-lasting legacy of deer on the plant community likely to establish in the next successional cycle after disturbance. Such legacy effects in succession also occur in response to invertebrate herbivory [75]. Aboveground, the major effect of deer was suppression of woody plants, which represents a different impact on succession. This aboveground effect was not a consequence of seedbank changes, but rather a direct effect of deer browsing.

Our findings highlight the different timescales over which successional processes occur. Some consequences of field abandonment occurred rapidly, whereas others occurred gradually over the entire period of the study. Our findings also illustrate the importance of considering changes in the soil seedbank, which are not always clearly reflected in the aboveground plant community. Lastly, our findings demonstrate multiple effects of deer on abandoned agricultural field plant communities. From a land management perspective, these effects vary in desirability. For instance, an increase in diversity could be desirable, whereas a weakening of competition against invasive species could be undesirable. This study contributes to the growing body of research identifying long-term, context-specific, and multidimensional impacts of large mammalian herbivores on early- to mid-successional plant communities.

## Supporting information

S1 Appendix Woody species observed in seedbank germination assay between 2005 and 2021. (DOCX)

S2 Appendix Percentage of species that were (A) biennial/perennial and (B) native in a greenhouse germination assay. Mean ± standard error across six blocks, alongside fitted model described in the text.
(DOCX)

S3 Appendix Indicator species analysis across all years. (DOCX)

S4 Appendix Effects of deer on aboveground plant communities between 2019 and 2022. (DOCX)

## References

[1] Davidson DW. The effects of herbivory and granivory on terrestrial plant succession. Oikos. 1993;68(1):23. doi: 10.2307/3545305

[2] De Jager NR, Drohan PJ, Miranda BM, Sturtevant BR, Stout SL, Royo AA, et al. Simulating ungulate herbivory across forest landscapes: a browsing extension for LANDIS-II. Ecological Modelling. 2017;350:11–29. doi: 10.1016/j.ecolmodel.2017.01.014

[3] De Vriendt L, Lavoie S, Barrette M, Tremblay J. From delayed succession to alternative successional trajectory: How different moose browsing pressures contribute to forest dynamics following clear‐cutting. J Veget Sci. 2020;32(1). doi: 10.1111/jvs.12945

[4] Hidding B, Tremblay J-P, Côté SD. A large herbivore triggers alternative successional trajectories in the boreal forest. Ecology. 2013;94(12):2852–60. doi: 10.1890/12-2015.1 24597230

[5] Lorentzen Kolstad A, Austrheim G, Solberg EJ, De Vriendt L, Speed JDM. Pervasive moose browsing in boreal forests alters successional trajectories by severely suppressing keystone species. Ecosphere. 2018;9(10). doi: 10.1002/ecs2.2458

[6] Petersson L, Salk C, Jensen D, Thor G, Ohkubo T. Long‐term deer exclusion releases dwarf bamboo, reducing vascular plant diversity. Appl Veget Sci. 2025;28(1). doi: 10.1111/avsc.70018

[7] Sitters J, Bakker ES, Veldhuis MP, Veen GF, Olde Venterink H, Vanni MJ. The stoichiometry of nutrient release by terrestrial herbivores and its ecosystem consequences. Front Earth Sci. 2017;5. doi: 10.3389/feart.2017.00032

[8] Popma JMA, Nadelhoffer KJ. Deer browsing effects on temperate forest soil nitrogen cycling shift from positive to negative across fertility gradients. Can J For Res. 2020;50(12):1281–8. doi: 10.1139/cjfr-2020-0036

[9] Sitters J, Olde Venterink H. Stoichiometric impact of herbivore dung versus urine on soils and plants. Plant Soil. 2021;462(1–2):59–65. doi: 10.1007/s11104-021-04960-7

[10] Williams SC, Ward JS. Exotic seed dispersal by white-tailed deer in southern Connecticut. Natural Areas Journal. 2006;26(4):383–90. doi: 10.3375/0885-8608(2006)26[383:esdbwd]2.0.co;2

[11] Bartuszevige AM, Endress BA. Do ungulates facilitate native and exotic plant spread?. J Arid Environ. 2008;72(6):904–13. doi: 10.1016/j.jaridenv.2007.11.007

[12] Guiden PW. Spatial heterogeneity in white-tailed deer activity increases seed dispersal of shade-intolerant plants near forest edges in fragmented forests. J Torrey Botan Soc. 2017;144(4):371–8. doi: 10.3159/torrey-d-16-00045.1

[13] Lepková B, Horčičková E, Vojta J. Endozoochorous seed dispersal by free-ranging herbivores in an abandoned landscape. Plant Ecol. 2018;219(9):1127–38. doi: 10.1007/s11258-018-0864-9

[14] Maron JL, Baer KC, Angert AL. Disentangling the drivers of context‐dependent plant–animal interactions. J Ecol. 2014;102(6):1485–96. doi: 10.1111/1365-2745.12305

[15] Eskelinen A, Jessen M-T, Bahamonde HA, Bakker JD, Borer ET, Caldeira MC, et al. Herbivory and nutrients shape grassland soil seed banks. Nat Commun. 2023;14(1):3949. doi: 10.1038/s41467-023-39677-x 37402739 PMC10319882

[16] Xu T, Cornwell W, Wang L, Wijas B, Liu C, Yuan Z, et al. Impacts of wild herbivores on soil seed banks are explained by precipitation conditions in protected areas across semi‐arid to arid regions. J Appl Ecol. 2024;61(12):2946–58. doi: 10.1111/1365-2664.14803

[17] Shinoda Y, Akasaka M. Interaction exposure effects of multiple disturbances: plant population resilience to ungulate grazing is reduced by creation of canopy gaps. Sci Rep. 2020;10(1):1802. doi: 10.1038/s41598-020-58672-6 32020019 PMC7000668

[18] Hanberry BB. Addressing regional relationships between white-tailed deer densities and land classes. Ecol Evol. 2021;11(19):13570–8. doi: 10.1002/ece3.8084 34646490 PMC8495829

[19] McShea WJ. Ecology and management of white-tailed deer in a changing world. Ann N Y Acad Sci. 2012;1249:45–56. doi: 10.1111/j.1749-6632.2011.06376.x 22268688

[20] Warren RJ. Deer overabundance in the USA: recent advances in population control. Anim Prod Sci. 2011;51(4):259. doi: 10.1071/an10214

[21] Côté SD, Rooney TP, Tremblay J-P, Dussault C, Waller DM. Ecological impacts of deer overabundance. Annu Rev Ecol Evol Syst. 2004;35:113–47.

[22] Rooney TP, Waller DM. Direct and indirect effects of white-tailed deer in forest ecosystems. Forest Ecol Manag. 2003;181(1–2):165–76. doi: 10.1016/s0378-1127(03)00130-0

[23] Waller DM, Alverson WS. The white-tailed deer: a keystone herbivore. Wildlife Soc Bulletin. 1997;25:217–26.

[24] Augustine DJ, McNaughton SJ. Ungulate effects on the functional species composition of plant communities: herbivore selectivity and plant tolerance. J Wildlife Manag. 1998;62(4):1165. doi: 10.2307/3801981

[25] Pickering JK, Bradstreet MSW, Norris DR. Less is more: vegetation changes coincide with white‐tailed deer suppression over thirty years. Wildlife Monog. 2024;214(1). doi: 10.1002/wmon.1081

[26] Russell FL, Zippin DB, Fowler NL. Effects of White-tailed Deer (Odocoileus virginianus) on plants, plant populations and communities: a review. Am Midland Natur. 2001;146(1):1–26. doi: 10.1674/0003-0031(2001)146[0001:eowtdo]2.0.co;2

[27] Russell MB, Woodall CW, Potter KM, Walters BF, Domke GM, Oswalt CM. Interactions between white-tailed deer density and the composition of forest understories in the northern United States. Forest Ecol Manag. 2017;384:26–33. doi: 10.1016/j.foreco.2016.10.038

[28] Hanberry BB, Abrams MD. Does white-tailed deer density affect tree stocking in forests of the Eastern United States?. Ecol Process. 2019;8(1). doi: 10.1186/s13717-019-0185-5

[29] Lesser MR, Dovciak M, Wheat R, Curtis P, Smallidge P, Hurst J, et al. Modelling white-tailed deer impacts on forest regeneration to inform deer management options at landscape scales. Forest Ecol Manag. 2019;448:395–408. doi: 10.1016/j.foreco.2019.06.013

[30] Royo AA, Stout SL, deCalesta DS, Pierson TG. Restoring forest herb communities through landscape-level deer herd reductions: is recovery limited by legacy effects?. Biol Conserv. 2010;143(11):2425–34. doi: 10.1016/j.biocon.2010.05.020

[31] Sabo AE, Frerker KL, Waller DM, Kruger EL. Deer‐mediated changes in environment compound the direct impacts of herbivory on understorey plant communities. J Ecol. 2017;105(5):1386–98. doi: 10.1111/1365-2745.12748

[32] Dobson A, Bowe A, Nuzzo V, Dávalos A, Fahey T, Blossey B. Individual and combined effects of non‐native earthworms and native white‐tailed deer on understorey plant survival, growth and reproduction. J Ecol. 2024;112(5):1039–57. doi: 10.1111/1365-2745.14285

[33] Dölle M, Schmidt W. The relationship between soil seed bank, above‐ground vegetation and disturbance intensity on old‐field successional permanent plots. Appl Veget Sci. 2009;12(4):415–28. doi: 10.1111/j.1654-109x.2009.01036.x

[34] Plue J, Van Calster H, Auestad I, Basto S, Bekker RM, Bruun HH, et al. Buffering effects of soil seed banks on plant community composition in response to land use and climate. Global Ecol Biogeogr. 2020;30(1):128–39. doi: 10.1111/geb.13201

[35] Warr SJ, Thompson K, Kent M. Seed banks as a neglected area of biogeographic research: a review of literature and sampling techniques. Prog Phys Geo Earth Environ. 1993;17(3):329–47. doi: 10.1177/030913339301700303

[36] Kiss R, Deák B, Török P, Tóthmérész B, Valkó O. Grassland seed bank and community resilience in a changing climate. Restor Ecol. 2018;26(S2). doi: 10.1111/rec.12694

[37] Bossuyt B, Honnay O. Can the seed bank be used for ecological restoration? An overview of seed bank characteristics in European communities. J Veg Sci. 2008;19(6):875–84. doi: 10.3170/2008-8-18462

[38] DiTommaso A, Morris SH, Parker JD, Cone CL, Agrawal AA. Deer browsing delays succession by altering aboveground vegetation and belowground seed banks. PLoS One. 2014;9(3):e91155. doi: 10.1371/journal.pone.0091155 24608258 PMC3946751

[39] Levine CR, Winchcombe RJ, Canham CD, Christenson LM, Ronsheim ML. Deer impacts on seed banks and saplings in Eastern New York. Northeastern Nat. 2012;19(1):49–66. doi: 10.1656/045.019.0104

[40] Myers JA, Vellend M, Gardescu S, Marks PL. Seed dispersal by white-tailed deer: implications for long-distance dispersal, invasion, and migration of plants in eastern North America. Oecologia. 2004;139(1):35–44. doi: 10.1007/s00442-003-1474-2 14740288

[41] Tamura A. Potential of soil seed banks in the ecological restoration of overgrazed floor vegetation in a cool-temperate old-growth damp forest in eastern Japan. J Forest Res. 2015;21(1):43–56. doi: 10.1007/s10310-015-0509-y

[42] Beauchamp VB, Ghuznavi N, Koontz SM, Roberts RP. Edges, exotics and deer: the seed bank of a suburban secondary successional temperate deciduous forest. Appl Veget Sci. 2013;16(4):571–84. doi: 10.1111/avsc.12036

[43] Reed SP, Royo AA, Carson WP, Olmsted CF, Frelich LE, Reich PB. Multiple disturbances, multiple legacies: Fire, canopy gaps and deer jointly change the forest seed bank. J Ecol. 2024;113(2):353–70. doi: 10.1111/1365-2745.14459

[44] USDA NRCS. Web soil survey. Accessed 2023 December 6. https://websoilsurvey.sc.egov.usda.gov/App/HomePage.htm

[45] Northeast Regional Climate Center. CLIMOD 2. Accessed 2025 August 28. http://climod2.nrcc.cornell.edu/

[46] R Core Team. R: a language and environment for statistical computing. R Foundation for Statistical Computing; 2024.

[47] Leck MA, Leck CF. A ten-year seed bank study of old field succession in central New Jersey. J Torrey Bot Soc. 1998;125(1):11. doi: 10.2307/2997228

[48] Bekker RM, Verweij GL, Bakker JP, Fresco LFM. Soil seed bank dynamics in hayfield succession. Journal of Ecology. 2000;88(4):594–607. doi: 10.1046/j.1365-2745.2000.00485.x

[49] Grandin U. Short‐term and long‐term variation in seed bank/vegetation relations along an environmental and successional gradient. Ecography. 2001;24(6):731–41. doi: 10.1111/j.1600-0587.2001.tb00534.x

[50] Thompson K, Grime JP. Seasonal variation in the seed banks of herbaceous species in ten contrasting habitats. J Ecol. 1979;67(3):893. doi: 10.2307/2259220

[51] Begley-Miller DR, Hipp AL, Brown BH, Hahn M, Rooney TP. White-tailed deer are a biotic filter during community assembly, reducing species and phylogenetic diversity. AoB Plants. 2014;6:plu030. doi: 10.1093/aobpla/plu030 24916059 PMC4078168

[52] Batzli GO, Dejaco CE. White-tailed Deer (Odocoileus virginianus) facilitate the development of nonnative grasslands in Central Illinois. Am Midl Nat. 2013;170(2):323–34. doi: 10.1674/0003-0031-170.2.323

[53] Bowers MA. Influence of herbivorous mammals on an old-field plant community: years 1-4 after disturbance. Oikos. 1993;67(1):129. doi: 10.2307/3545103

[54] Goetsch C, Wigg J, Royo AA, Ristau T, Carson WP. Chronic over browsing and biodiversity collapse in a forest understory in Pennsylvania: Results from a 60 year-old deer exclusion plot. J Torrey Bot Soc. 2011;138(2):220–4. doi: 10.3159/torrey-d-11-00013.1

[55] Pendergast TH IV, Hanlon SM, Long ZM, Royo AA, Carson WP. The legacy of deer overabundance: long-term delays in herbaceous understory recovery. Can J For Res. 2016;46(3):362–9. doi: 10.1139/cjfr-2015-0280

[56] Horsley SB, Stout SL, deCalesta DS. White-tailed deer impact on the vegetation dynamics of a northern hardwood forest. Ecol Appl. 2003;13(1):98–118. doi: 10.1890/1051-0761(2003)013[0098:wtdiot]2.0.co;2

[57] Reed SP, Royo AA, Fotis AT, Knight KS, Flower CE, Curtis PS. The long‐term impacts of deer herbivory in determining temperate forest stand and canopy structural complexity. J Appl Ecol. 2021;59(3):812–21. doi: 10.1111/1365-2664.14095

[58] Royo AA, Collins R, Adams MB, Kirschbaum C, Carson WP. Pervasive interactions between ungulate browsers and disturbance regimes promote temperate forest herbaceous diversity. Ecology. 2010;91(1):93–105. doi: 10.1890/08-1680.1 20380200

[59] Simončič T, Bončina A, Jarni K, Klopčič M. Assessment of the long‐term impact of deer on understory vegetation in mixed temperate forests. J Veg Sci. 2019;30(1):108–20. doi: 10.1111/jvs.12702

[60] Urbanek RE, Nielsen CK, Glowacki GA, Preuss TS. Effects of white-tailed deer (*Odocoileus virginianus* Zimm.) herbivory in restored forest and savanna plant communities. Am Midl Nat. 2012;167(2):240–55. doi: 10.1674/0003-0031-167.2.240

[61] Averill KM, Mortensen DA, Smithwick EAH, Post E. Deer feeding selectivity for invasive plants. Biol Invasions. 2016;18(5):1247–63. doi: 10.1007/s10530-016-1063-z

[62] Williams SC, Ward JS, Ramakrishnan U. Endozoochory by white-tailed deer (Odocoileus virginianus) across a suburban/woodland interface. Forest Ecol Manag. 2008;255(3–4):940–7. doi: 10.1016/j.foreco.2007.10.003

[63] Baltzinger C, Karimi S, Shukla U. Plants on the move: hitch-hiking with ungulates distributes diaspores across landscapes. Front Ecol Evol. 2019;7. doi: 10.3389/fevo.2019.00038

[64] Yang X, Baskin CC, Baskin JM, Pakeman RJ, Huang Z, Gao R, et al. Global patterns of potential future plant diversity hidden in soil seed banks. Nat Commun. 2021;12(1):7023. doi: 10.1038/s41467-021-27379-1 34857747 PMC8639999

[65] Schmit JP, Matthews ER, Brolis A. Effects of culling white-tailed deer on tree regeneration and Microstegium vimineum, an invasive grass. Forest Ecol Manag. 2020;463:118015. doi: 10.1016/j.foreco.2020.118015

[66] Popay I. *Rosa multiflora* (multiflora rose). CABI Compendium. CABI Publishing; 2013. doi: 10.1079/cabicompendium.47824

[67] Bowden RD, Caylor A, Hemmelgarn G, Kresse M, Martin A, Althouse M. Prescribed browsing by goats shows promise in controlling multiflora rose in a deciduous forest at the erie national wildlife refuge in northwestern Pennsylvania. Natural Areas J. 2022;42(3). doi: 10.3375/21-30

[68] Wilson L, Davison J, Smith E. Grazing and browsing guidelines for invasive rangeland weeds. In: Launchbaugh K, ed. Targed grazing: a natural approach to vegetation management. American Sheep Industry Association; 2006. 142–67.

[69] Atwood EL. White-tailed deer foods of the United States. J Wildlife Manag. 1941;5(3):314. doi: 10.2307/3795797

[70] Wade GL, Mengak MT. Deer-tolerant ornamental plants. Circular 985. University of Georgia Cooperative Extension; 2010.

[71] McGarvey JC, Bourg NA, Thompson JR, McShea WJ, Shen X. Effects of twenty years of deer exclusion on woody vegetation at three life-history stages in a mid-atlantic temperate deciduous forest. Northeast Nat. 2013;20(3):451–68. doi: 10.1656/045.020.0301

[72] Ruzicka KJ, Groninger JW, Zaczek JJ. Deer browsing, forest edge effects, and vegetation dynamics following bottomland forest restoration. Restor Ecol. 2010;18(5):702–10. doi: 10.1111/j.1526-100x.2008.00503.x

[73] Hopfensperger KN. A review of similarity between seed bank and standing vegetation across ecosystems. Oikos. 2007;116(9):1438–48. doi: 10.1111/j.0030-1299.2007.15818.x

[74] Wang N, Jiao J-Y, Jia Y-F, Bai W-J, Zhang Z-G. Germinable soil seed banks and the restoration potential of abandoned cropland on the Chinese hilly-gullied loess plateau. Environ Manage. 2010;46(3):367–77. doi: 10.1007/s00267-010-9535-x 20694556

[75] Agrawal AA, Hastings AP, Maron JL. Evolution and seed dormancy shape plant genotypic structure through a successional cycle. Proc Natl Acad Sci U S A. 2021;118(34):e2026212118. doi: 10.1073/pnas.2026212118 34400497 PMC8403902

