# Supplementary material for: Deer impact seedbanks and plant communities over 18 years of post-agricultural succession
Source: PLoS One. 2025 Dec 23;20(12):e0339466. doi: 10.1371/journal.pone.0339466 (PMC12725539; doi:10.1371/journal.pone.0339466)
Supplement: S1 Appendix — (DOCX) [file pone.0339466.s001.docx]

**Appendix S1.** Woody species observed in seedbank germination assay between 2005 and 2021.

| Common name | Scientific name | Growth form | Count in browsed control treatment | Count in exclosure treatment |
| --- | --- | --- | --- | --- |
| Box elder | *Acer negundo* L. | Tree | 0 | 1 |
| Eastern cottonwood | *Populus deltoides* W. Bartram ex Marshall | Tree | 12 | 14 |
| Staghorn sumac | *Rhus typhina* L. | Tree | 4 | 3 |
| Black locust | *Robinia pseudoacacia* L. | Tree | 1 | 0 |
| Black raspberry | *Rubus occidentalis* L. | Shrub | 6 | 5 |
| Riverbank grape | *Vitis riparia* Michx. | Woody vine | 0 | 1 |
