# Supplementary material for: Deer impact seedbanks and plant communities over 18 years of post-agricultural succession
Source: PLoS One. 2025 Dec 23;20(12):e0339466. doi: 10.1371/journal.pone.0339466 (PMC12725539; doi:10.1371/journal.pone.0339466)
Supplement: S2 Appendix — Mean ± standard error across six blocks, alongside fitted model described in the text. (DOCX) [file pone.0339466.s002.docx]

**
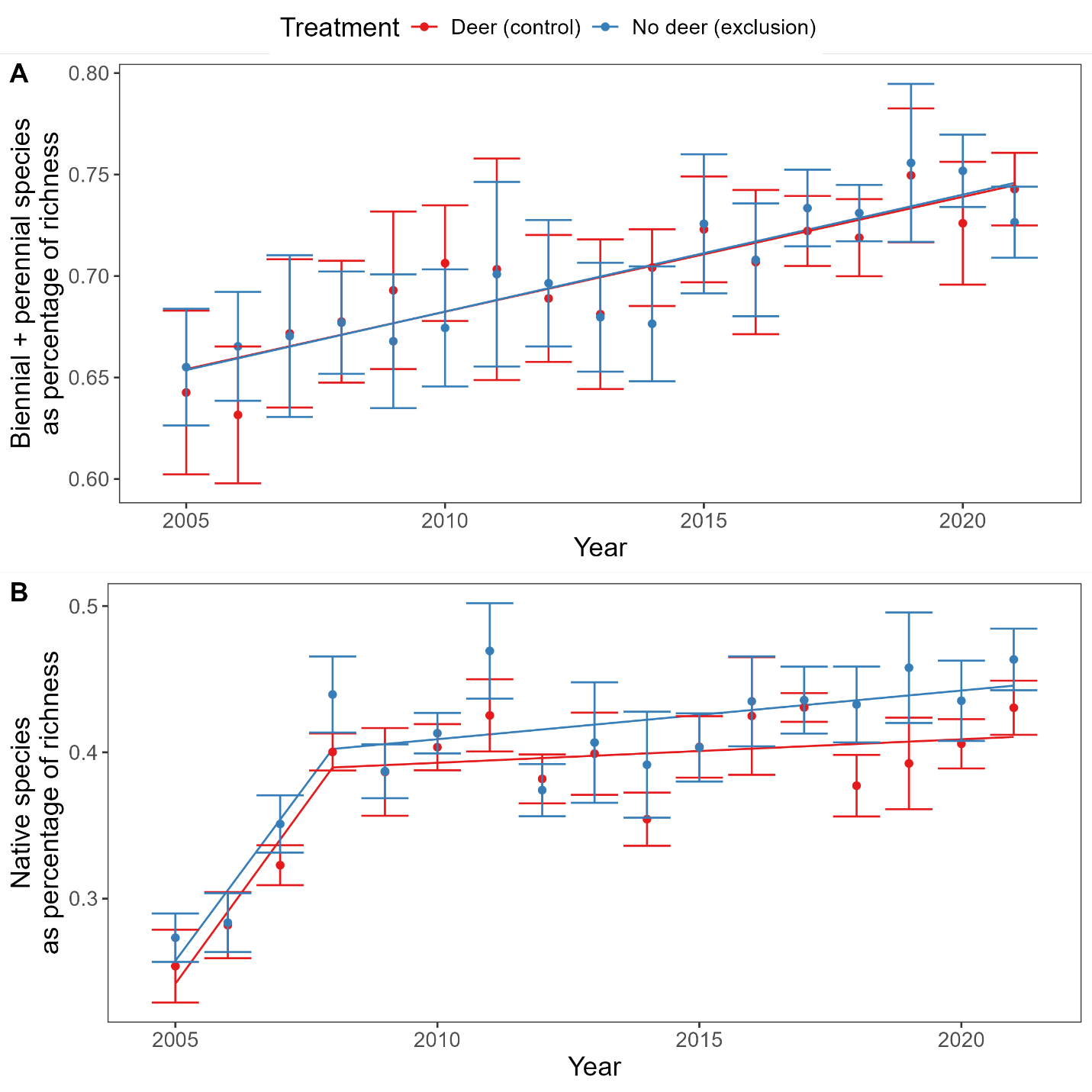
Appendix S2.** Percentage of species that were (A) biennial/perennial and (B) native in a greenhouse germination assay. Mean ± 1 standard error across six blocks, alongside fitted model described in the text.
