# Supplementary material for: Deer impact seedbanks and plant communities over 18 years of post-agricultural succession
Source: PLoS One. 2025 Dec 23;20(12):e0339466. doi: 10.1371/journal.pone.0339466 (PMC12725539; doi:10.1371/journal.pone.0339466)
Supplement: S3 Appendix — (DOCX) [file pone.0339466.s003.docx]

**Appendix S3.** Indicator species analysis across all years.

| Common name | Scientific name | Life cycle^1^ | Native^2^ | A^3^ | B^4^ | Stat^5^ | P |
| --- | --- | --- | --- | --- | --- | --- | --- |
| Deer (control treatment) | | | | | | | |
| Grass-leaved stitchwort | *Stellaria graminea* L. | P | I | 0.89 | 0.52 | 0.68 | 0.19 |
| Orchardgrass | *Dactylis glomerata* L. | P | I | 0.83 | 0.09 | 0.27 | 0.06 |
| Large crabgrass | *Digitaria sanguinalis* (L.) Scop. | A | I | 0.83 | 0.06 | 0.22 | 0.38 |
| Perennial sowthistle | *Sonchus arvensis* L. | P | I | 0.82 | 0.04 | 0.18 | 0.74 |
| Catchweed bedstraw | *Galium aparine* L. | A | I | 1.00 | 0.03 | 0.17 | 0.25 |
| Yellow avens | *Geum aleppicum* Jacq. | P | N | 0.80 | 0.02 | 0.13 | 0.48 |
| Spreading atriplex | *Atriplex patula* S. Watson | A | I | 1.00 | 0.01 | 0.10 | 1.00 |
| Greater straw sedge | *Carex normalis* Mack. | P | N | 1.00 | 0.01 | 0.10 | 1.00 |
| Goosegrass | *Eleusine indica* (L.) Gaertn. | A | I | 1.00 | 0.01 | 0.10 | 1.00 |
| Tall fescue | *Lolium arundinaceum* (Schreb.)  S.J. Darbyshire | P | I | 1.00 | 0.01 | 0.10 | 1.00 |
| Low cudweed | *Gnaphalium uliginosum* L. | A | I | 1.00 | 0.01 | 0.10 | 1.00 |
| Black locust | *Robinia pseudoacacia* L. | P | N | 1.00 | 0.01 | 0.10 | 1.00 |
| Common groundsel | *Senecio vulgaris* L. | A | I | 1.00 | 0.01 | 0.10 | 1.00 |
| Heath aster | *Symphyotrichum ericoides*  (L.) G.L. Nesom | P | N | 1.00 | 0.01 | 0.10 | 1.00 |
| No deer (exclosure treatment) | | | | | | | |
| Common teasel | *Dipsacus fullonum* L. | B | I | 0.83 | 0.29 | 0.50 | 0.14 |
| Hemp nettle | *Galeopsis tetrahit* L. | A | I | 0.95 | 0.13 | 0.35 | 0.25 |
| Oakleaf goosefoot | *Chenopodium glaucum* L. | A | I | 0.77 | 0.12 | 0.30 | 0.33 |
| Clearweed | *Pilea pumila* (L.) A. Gray | A | N | 0.93 | 0.06 | 0.23 | 1.00 |
| Field pennycress | *Thlaspi arvense* L. | A | I | 0.87 | 0.06 | 0.23 | 0.48 |
| Common speedwell | *Veronica officinalis* L. | A | I | 0.79 | 0.04 | 0.18 | 0.53 |
| Black medic | *Medicago lupulina* L. | A | I | 0.93 | 0.03 | 0.17 | 0.27 |
| Dark green bulrush | *Scirpus atrovirens* Willd. | P | N | 0.90 | 0.03 | 0.16 | 1.00 |
| Yellow foxtail | *Setaria pumila* (Poir.)  Roem. & Schult. | A | I | 0.73 | 0.03 | 0.15 | 0.27 |
| Devil's beggarsticks | *Bidens frondosa* L. | A | N | 1.00 | 0.02 | 0.14 | 1.00 |
| Hedge bindweed | *Calystegia sepium* (L.) R. Br. | P | I | 1.00 | 0.02 | 0.14 | 1.00 |
| Arrow-leaved tearthumb | *Polygonum sagittatum* L. | A | N | 1.00 | 0.02 | 0.14 | 1.00 |
| Heart-leaved aster | *Symphyotrichum cordifolium*  (L.) G.L. Nesom | P | N | 1.00 | 0.02 | 0.14 | 0.51 |
| Common purslane | *Portulaca oleracea* L. | A | I | 0.95 | 0.02 | 0.14 | 0.52 |
| Roughstalk bluegrass | *Poa trivialis* L. | P | I | 0.80 | 0.02 | 0.13 | 0.50 |
| Box elder | *Acer negundo* L. | P | N | 1.00 | 0.01 | 0.10 | 1.00 |
| Chicory | *Cichorium intybus* L. | P | I | 1.00 | 0.01 | 0.10 | 1.00 |
| Yellow hawkweed | *Pilosella caespitosa* (Dumort)  P.D.Sell & C.West | P | I | 1.00 | 0.01 | 0.10 | 1.00 |
| Nodding bulrush | *Scirpus pendulus* Muhl. | P | N | 1.00 | 0.01 | 0.10 | 1.00 |
| Gray goldenrod | *Solidago nemoralis* Aiton | P | N | 1.00 | 0.01 | 0.10 | 1.00 |
| Purple-stemmed aster | *Symphyotrichum puniceum*  (L.) Á. Löve & D. Löve | P | N | 1.00 | 0.01 | 0.10 | 1.00 |
| Riverbank grape | *Vitis riparia* Michx. | P | N | 1.00 | 0.01 | 0.10 | 1.00 |

^1^Annual (A), biennial (B), or perennial (C), according to the most common life cycle in New York, USA.

^2^Native (N) or introduced (I) to New York, USA

^3^Specificity. If the listed species is present at a site, specificity is the probability that this site is in the indicated treatment group.

^4^Fidelity or sensitivity. If a site is in the indicated treatment group, fidelity is the probability of finding the listed species.

^5^The test statistic is the association index IndVal.g.
