# Supplementary material for: Deer impact seedbanks and plant communities over 18 years of post-agricultural succession
Source: PLoS One. 2025 Dec 23;20(12):e0339466. doi: 10.1371/journal.pone.0339466 (PMC12725539; doi:10.1371/journal.pone.0339466)
Supplement: S4 Appendix — (DOCX) [file pone.0339466.s004.docx]

**Appendix S4.** Effects of deer on aboveground plant communities between 2019 and 2022.

| Response | Year | Exclosure treatment  F (numerator df, denominator df) | P |
| --- | --- | --- | --- |
| Plant cover | 2019 | 3.0 (1, 10) | 0.11 |
|  | 2022 | 6.4 (1, 5) | 0.053 |
| Species richness | 2019 | 0.87 (1, 5) | 0.39 |
|  | 2022 | 0.00 (1, 5) | 1.0 |
| Shannon-Wiener diversity | 2019 | 0.045 (1, 5) | 0.84 |
|  | 2022 | 0.25 (1, 5) | 0.64 |
| Biennial/perennial species as percentage of richness | 2019 | 1.3 (1, 10) | 0.28 |
|  | 2022 | 2.5 (1, 5) | 0.18 |
| Native species as percentage of richness | 2019 | 4.6 (1, 5) | 0.086 |
|  | 2022 | 4.5 (1, 5) | 0.089 |
| Goldenrod biomass | 2019 | 0.0052 (1, 5) | 0.95 |
| Goldenrod height | 2021 | 1.6 (1, 5) | 0.26 |
